# Wernicke’s Encephalopathy Masquerading as an Acute Cerebellar Stroke

**DOI:** 10.7759/cureus.82826

**Published:** 2025-04-23

**Authors:** Nidha Shapoo, Noella Boma

**Affiliations:** 1 Internal Medicine, New York Medical College, Metropolitan Hospital Center, New York, USA

**Keywords:** alcohol use disorder, reversible syndrome, stroke mimics, thiamine deficiency, wernicke’s encephalopathy

## Abstract

Wernicke’s encephalopathy (WE) is an acute neurological emergency resulting from severe thiamine deficiency, frequently mimicking acute cerebellar stroke due to its abrupt onset and neurological manifestations. Early recognition is crucial to prevent severe complications. Here, we report the case of a 52-year-old male with alcohol use disorder who presented with acute dizziness, vomiting, and gait instability. Neurological examination revealed left facial droop, dysarthria, and an ataxic gait with a positive Romberg sign. Given the presentation, an acute stroke was initially suspected (National Institute of Health Stroke Scale score: 4), but computed tomography and computed tomography angiography excluded vascular pathology. Magnetic resonance imaging findings demonstrated bilateral thalamic and periaqueductal hyperintensities, confirming WE. Intravenous thiamine administration led to rapid clinical improvement, and the patient was discharged on oral thiamine in a stable condition. WE remains underdiagnosed due to its variable presentation. While nystagmus is a hallmark sign, ataxia is also significant. Given its reversibility, clinicians should maintain a high index of suspicion, especially in patients with alcohol use disorder presenting with cerebellar signs. Prompt thiamine administration can prevent severe morbidity and mortality.

## Introduction

Wernicke’s encephalopathy (WE) is an acute, reversible neurological syndrome caused by severe thiamine deficiency, with a reported prevalence of 0.4% to 2.8% [1,2]. It is a medical emergency characterized by a classical triad of ophthalmoplegia, ataxia, and confusion, though observed in only one-third of cases [1,2]. WE is most commonly associated with chronic alcohol use disorder, but can also occur in patients with chronic malnutrition, malignancy, hemodialysis, and human immunodeficiency virus [3]. If untreated, WE may progress to Korsakoff psychosis and irreversible neurological damage, including death [4].

Stroke remains the second leading cause of mortality worldwide and a significant cause of disability. Cerebellar stroke, a rare subtype of acute ischemic stroke due to posterior circulation infarction, accounts for approximately 3% of all ischemic strokes, with an estimated annual incidence of 20,000 cases in the United States. The mortality rate of cerebellar strokes ranges from 9% to 39%, nearly double that of cerebral strokes (12.5%) [5,6].

Stroke mimics, defined as conditions that produce focal neurological deficits without vascular involvement, can delay the diagnosis of reversible conditions due to the similarity of their symptoms. Stroke mimics occur in up to 30% of cases presenting with acute neurological deficits [7]. Given its neurological presentation, WE can resemble an acute cerebellar stroke [8]. As WE is a clinical diagnosis, maintaining a high index of suspicion in at-risk populations, even in the absence of the entire triad, is essential for early recognition and treatment [2,3].

Here, we present the case of a 52-year-old male whose presentation initially raised concerns for an acute cerebellar stroke, leading to a stroke alert.

## Case presentation

A 52-year-old male with a medical history of hypertension, diabetes mellitus, and alcohol use disorder (three beers per day for 15 years) presented with the sudden onset of dizziness, vomiting, and gait instability. The patient presented six hours after symptom onset. Upon admission, the patient was conscious and oriented but inattentive, with a heart rate of 88 beats per minute and blood pressure of 180/89 mmHg.

Neurological examination revealed equal and reactive pupils with no nystagmus. However, the patient exhibited a left facial droop, dysarthria, and an ataxic gait with a positive Romberg sign. The motor and sensory function was intact, deep tendon reflexes were normal, and bilateral plantar responses were flexor. The National Institute of Health Stroke Scale score was 4, indicating a favorable clinical outcome and a high likelihood of functional independence [9].

**Figure 1:**
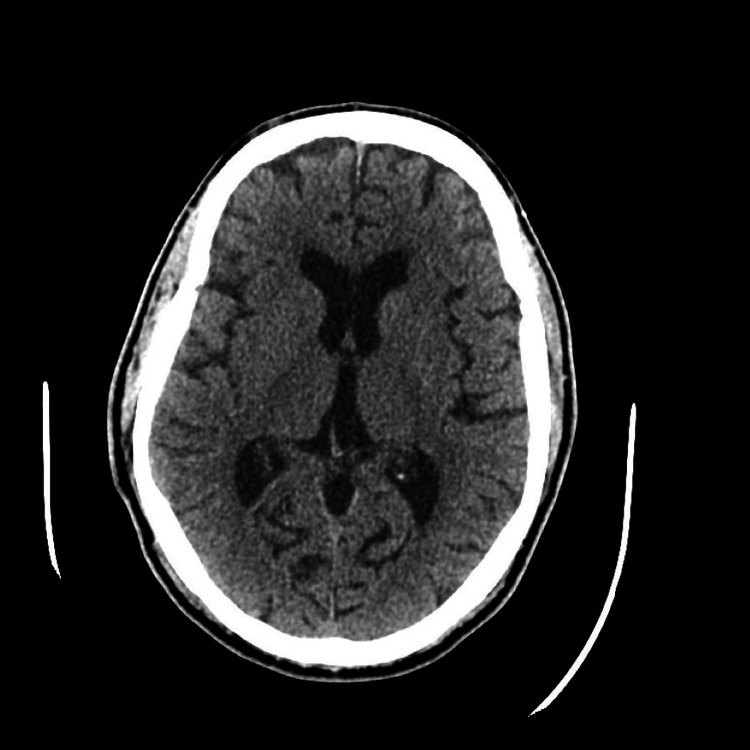
Computed tomography of the head revealing chronic white matter microangiopathy with no arterial occlusion, obstructive stenosis, or aneurysm.

**Figure 2:**
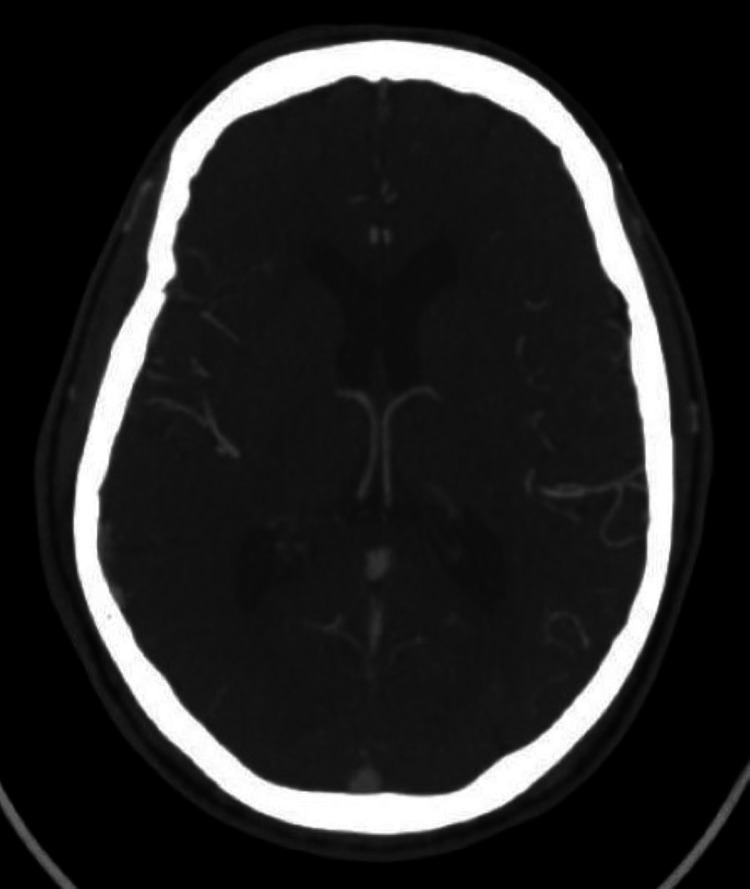
Computed tomography angiography of the head revealing chronic white matter microangiopathy with no arterial occlusion, obstructive stenosis, or aneurysm.

A non-contrast computed tomography (CT) of the head and CT angiography of the head and neck revealed chronic white matter microangiopathy without arterial occlusion, obstructive stenosis, or aneurysm (Figures 1, 2).

Laboratory investigations revealed a hemoglobin level of 13.2 g/dL (reference range: 12-16 g/dL), a platelet count of 77,000/µL (reference range: 150,000-450,000/µL), and a blood glucose level of 291 mg/dL (reference range: 74-109 mg/dL). Renal and liver function tests, serum electrolytes, and serum ethanol levels (<10 mg/dL) were unremarkable. The urine toxicology screen was negative.

**Figure 3:**
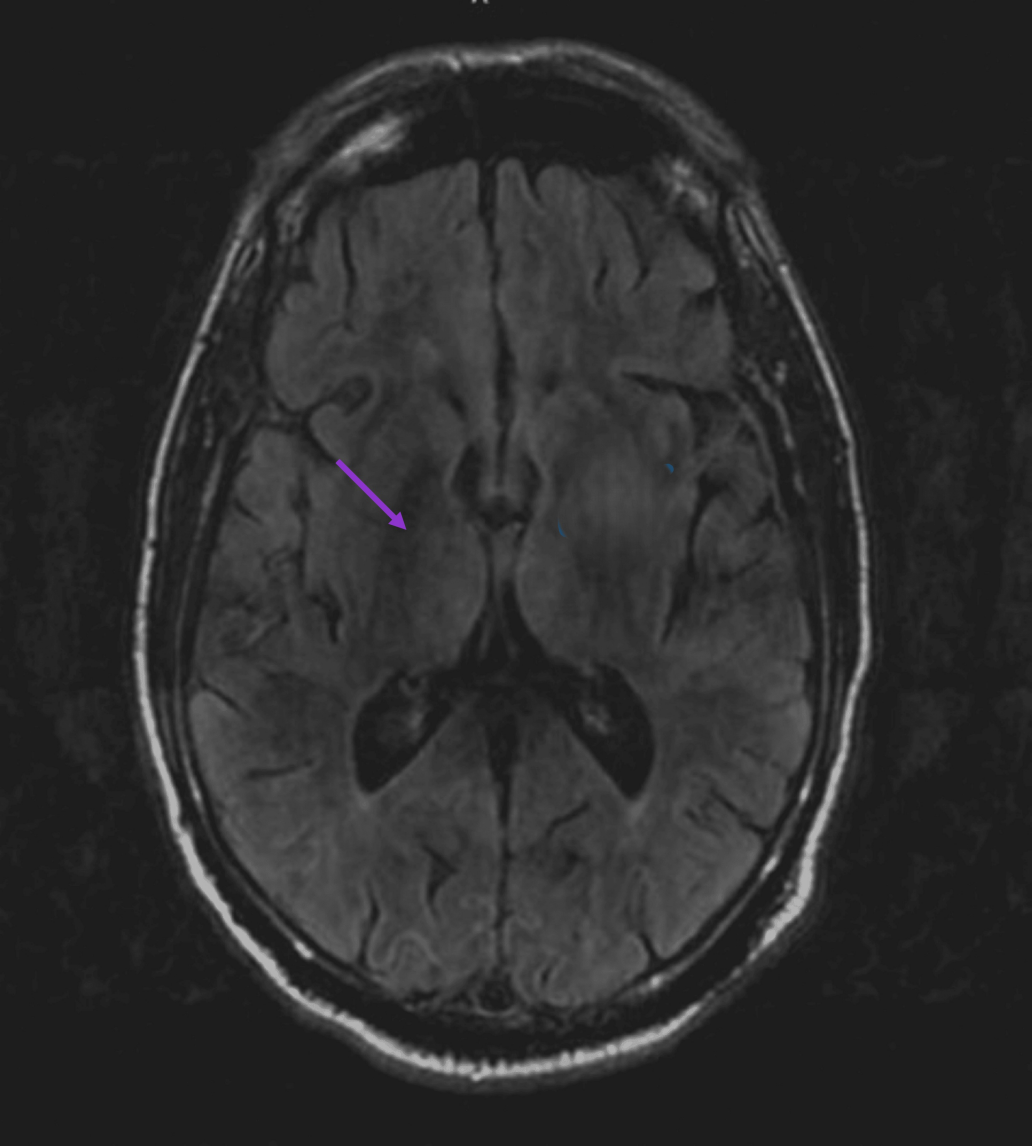
Magnetic resonance imaging of the brain showing symmetrical abnormal fluid-attenuated inversion recovery hyperintensity in the medial bilateral thalami.

**Figure 4:**
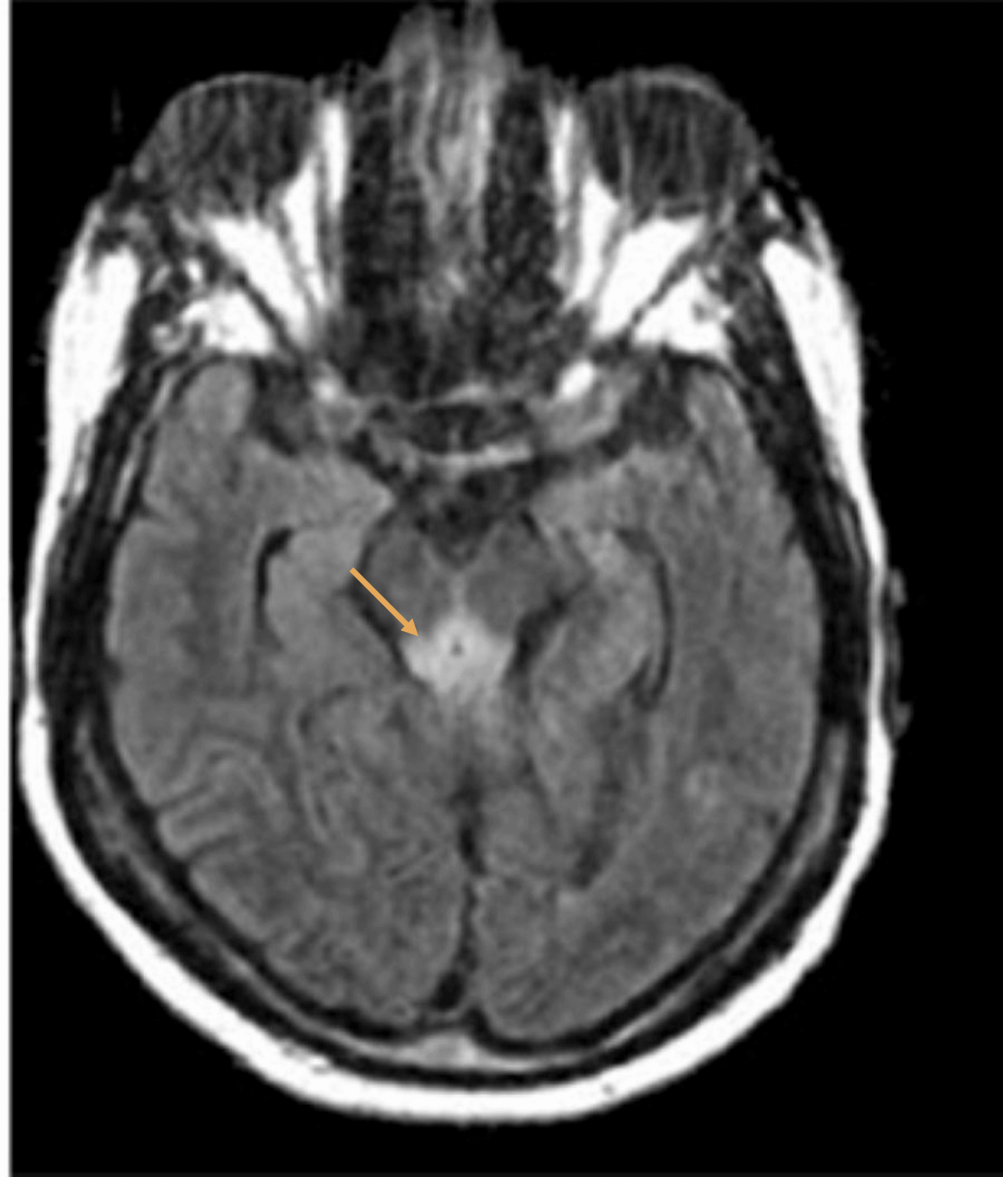
Magnetic resonance imaging of the brain revealing symmetrical abnormal fluid-attenuated inversion recovery hyperintensity within the periaqueductal gray matter.

Magnetic resonance imaging (MRI) of the brain demonstrated symmetrical fluid-attenuated inversion recovery hyperintensities in the bilateral thalami, periaqueductal gray matter, and the adjacent third ventricle, characteristic of WE. No infarct or hemorrhage was identified (Figures 3, 4).

The patient was promptly treated with intravenous thiamine 500 mg three times daily for two days, followed by 250 mg daily for five days. His symptoms improved significantly, with the resolution of dizziness and vomiting. He regained the ability to walk unassisted without ataxia. The patient was transitioned to oral thiamine and discharged in stable condition.

## Discussion

Chronic heavy alcohol use depletes thiamine stores, disrupting the Krebs cycle and the pentose phosphate pathway, resulting in decreased ATP production and increased oxidative stress. Toxic metabolites, such as lactate, alanine, and glutamate, accumulate, resulting in vasogenic and cytotoxic edema. The most affected brain regions in WE include the thalami (85%), periaqueductal area (59%-65%), and mammillary bodies (~45%) [10].

The classical triad of WE is infrequently observed, leading to underdiagnosis. Mental status changes, including confusion, inattention, and memory disturbances, are the most common symptoms, with delirium being a consistent clinical feature [11]. Ocular abnormalities, particularly horizontal nystagmus, and varying degrees of gait ataxia further complicate the diagnosis [2,3].

Cerebellar stroke presents acutely with dizziness, vertigo, ataxia, and impaired gait, necessitating rapid intervention to prevent long-term disability and mortality [5]. Due to heightened awareness and rapid management protocols for stroke, WE is often overlooked.

MRI has a sensitivity of ~50% and specificity of ~90% for WE diagnosis, though findings may normalize within days of thiamine initiation. Mammillary body involvement is considered pathognomonic [5].

Roçi et al. described a 63-year-old male who presented with left-sided weakness, dizziness, and confusion, initially suspected of transient ischemic attack but later diagnosed with WE after an MRI revealed mammillary and hypothalamic involvement, consistent with alcohol-induced thiamine deficiency [12]. Similarly, Bhan et al. reported a 51-year-old male presenting with dysarthria and unilateral facial weakness, mimicking acute stroke, but ultimately diagnosed with WE [8]. Both cases were successfully treated with intravenous thiamine treatment.

Prompt administration of intravenous thiamine is crucial to prevent severe neurological sequelae and death. The recommended regimen consists of intravenous thiamine 500 mg three times daily for two days, followed by 250 mg intravenous/intramuscular for five days, along with other B vitamins. Maintenance therapy with oral thiamine (100 mg daily) is essential after discharge to prevent recurrence [5].

## Conclusions

WE remains an underdiagnosed yet reversible neurological emergency. This case highlights the need for high clinical suspicion, particularly in patients with alcohol use disorder presenting with acute cerebellar symptoms. Prompt thiamine supplementation can lead to significant recovery, preventing severe morbidity and mortality. As stroke mimics can significantly impact clinical decision-making, comprehensive neurological assessment and imaging are essential to ensure accurate diagnosis and appropriate treatment.
